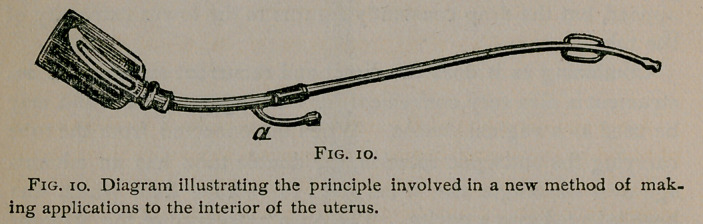# A New Pump and Its Uses in Medicine

**Published:** 1889-03

**Authors:** Wm. B. Gilmer

**Affiliations:** Atlanta, Georgia


### A NEW PUMP AND ITS USES IN MEDICINE.

BY WM. B. GILMER, M. D., ATLANTA, GEORGIA.

During the greater part of the twelve months ending last June, I was engaged in assisting Dr. Bozeman of New York, in the preparation for publication, among other things, of an account of his new operation of Kolpo-uretero-cystomy, and the description of his instruments for collecting the urine escaping through the permanent opening of the vesico-vaginal septum.* The results obtained in the treatment of cystitis and pyelitis by his methods, seemed to me to be brilliant, but the drainage secured by the use of his drains was not perfect, and, although the evil consequences of incontinence of urine were in part prevented, the bed and the patient's clothing in some of the cases, were more or less constantly saturated with urine. Becoming, through my connections with the work, deeply interested in and anxious for the success of the operation, I began to try to remedy this defect by improving the drainage instruments. I hoped to so modify them as to be able to make an opening in the bladder, and to secure all the advantages which are derived from physiological rest of the urinary organs, while at the same time to promise the patient freedom from the discomfort arising from incontinence of urine.

*Trans. Ninth Internat. Med. Congress and Amer. Jour. Med. Sciences, for March and April, 1888.

I did not succeed, but the experiments begun with this object in view, and extended over more than a year, have led step by step to other results, which will, I believe, prove equally useful and capable of more general application. In order, therefore, that the reader may begin as I did, it is necessary to go over, as briefly as possible, what had been done by Bozeman and others in the palliative treatment of incontinence of urine, the result of vesico-vaginal fistula.

The simplest method of preventiug the continuous escape of the urine, is to place in the vagina some substance which will fill its cavity and obturate the fistulous opening. In this way, so long as the plug is in position, the urine may be more or less entirely confined in the bladder. We find in the writings of the early writers accounts of patients, who, when they wished to be more tidy than usual, packed the vagina with linen rags. In Churchill's System of Midwifery, there are a number of instruments described which are intended to act in this manner. One of them consists of a caoutchouc bottle, covered at one part of its surface by an oblong piece of sponge, another of a cylinder of sponge enclosed in a bladder.

Skene has described instruments* of his own invention, which, while they depend for their efficiency upon the close contact of the vaginal mucuous membrane with their surfaces, introduce a new principle of action. One of them is a hollow sphere, with perforations leading into its interior, distributed over its surface. At one part, there is a nozzle which is connected by a soft rubber tube with a urinal attached to the thigh near the knee. The important difference between this instrument and the simple vaginal plug is, that the urine is no longer pent up in the bladder, but the accurate contact of the mucuous membrane tending to prevent its escape in any other direction, the fluid passes through the perforations in the instrument, and thence out through the rubber tube away from the body.

The instruments of Bozeman, which appeared later, present several points of similarity with those of Skene. They are hollow vessels of various shapes, pierced by openings for the en-

*Diseases of the Bladder and Uretha in Women trance of urine, and connected by a tube with a rubber bag. They differ from the perforated sphere of Skene in shape, and the perforations are confined to the upper or vesical surface, which also is made concave. The concavity serves to prevent rotations of the instrument in the vagina, favors the passage of urine through the perforations, and increases the extent and closeness of contact of the mucous membrane of this part. The various shapes given to the instruments by Dr. Bozeman are intended to adapt them, in addition to the drainage of the bladder, to the performance of two other functions, dilatation of cicatricial narrowing of the vagina, and support of the uterus. The "Drainage dilators" consist of hollow cylinders with rounded extremities. The "Drainage supports" are flattened cylinders, contracted anteriorly where they are connected with the rubber tube, but having broad and rounded posterior extremities, which rest beneath the cervix uteri.

For the efficient action of these various forms of instruments, there is one essential condition common to all; they must fit the vagina. In proportion as the vagina retains its flexibility, and the mucous membrane is closely applied to the surface of the instrument, the greater the quantity of urine collected. When the uterus is fixed, however, or the presence of cicatricial tissue causes rigidity or irregularity of the vaginal canal, a fit can only be secured by the use of a special instrument made for each case, and then only imperfectly. Tenderness about the uterus, ovaries, or any of the pelvic organs, by limiting the size of the instrument that can be tolerated, diminishes the closeness of contact of the mucous membrane with its surface. Indeed, this sensitiveness may be so great as to prevent, at least for a time, the use of any instrument, however small. In addition to these difficulties, others of less importance are encountered. Lacerations of the perinæum, and redundancy, or a tendency to prolapse of the vaginal walls, render the maintenance of the drain in position difficult. In the recumbent position, the inclination of the instrument at about the angle of 35° to the horizon is unfavorable.* When the patient is in bed, another cause of failure is the resistance op-

* Bozeman on Chronic Pyelitis, *Journal Medical Sciences*, March, 1888.

posed to the entrance of urine into the urinal after it is partly filled.

From one or other of these causes, the drainage secured by the use of any of the instruments which have been described, is not perfect, and in some cases is very unsatisfactory.

When I began to study this subject, my attention was at first directed to the form of the drainage instruments. I accordingly made them of many shapes. I learned to mould them in gutta percha, and the models were sufficiently strong to be used for several days. The greater number of these instruments were tried by Dr. Bozeman in the case of a patient in his service at the Woman's Hospital. In consequence of fixation of the uterus and pelvic inflammation, the drainage, secured by the use of his drains, was very imperfect, and those which I made were equally unsatisfactory.

These experiments, which cost a great deal of time and labor, served only to teach me that complete success, at least in complicated cases, could not be attained by the use of any form of instrument, which depended for its efficiency upon the close apposition of the mucous membrane. It became evident that some force must be called into action to seize hold of the urine, raise it over the perinæum, and conduct it away from the body. Capillarity naturally suggests itself as available for this purpose. I accordingly made a few experiments with pieces of lamp-wicking, cloth, and other porous materials, dipping them in water and allowing the ends to hang over the outside of the vessel. By using as large a piece of lamp-wicking as could be conveniently employed in the vagina, I found that the urine could be raised a sufficient heighth to lift it over the perinæum, but not in sufficient quantity. Aside from this great difficulty, other objections were at once apparent; the urine would decompose and the salts of lime be deposited in the pores of the fabric.

Abandoning, therefore, lampwicking, but not the force of capillarity, I next thought of what seemed a more promising expedient; and let us study this latter with care, because, besides being curious in itself, there is involved in it much which became of use. Take two pieces of soft rubber tubing, one small and short, the other considerably longer and of such a size as to allow the smaller tube to fit loosely within it. When the tubes are placed one inside of the other, we have two surfaces very near together. Fluid will rise between them, in virtue of capillary attraction, to a height inversel} proportional to the distance which separates them. In practice I found that water could be raised an inch and a half. This is a sufficient height, because in the recumbent position the outlet of the vagina is about this much above the lowest part of the canal. When the tubes are arranged, as shown in

FIG. 1. A section of the Self-starting Syphon, showing the relative positions of the internal and external tubes. Between the descending limb of the tube and the glass, is also shown a section of the contrivance employed to make the water fill the tube.

figure 1, the water rises in virtue of capillarity, and slowly forms in drops at *a*, the end of the internal tube. These drops, descending like minute pistons, carry the air with them, a partial vacuum is gradually produced, and the water rises in the interior of the smaller tube as well as between the two surfaces instead of four or five drops a minute (less than the kidneys secrete), a stream, which quickly empties the glass, now flows through the tubes. We have started a syphon by capillary attraction.

I made a small cage of fine wire gauze in order to separate the walls of the vagina, and to form a cavity to hold urine analagous to the glass in the above experiment. The self-starting syphon was placed in the cage and, when the instrument was introduced, the tube hung down over the perinæum and dipped in a shallow cup placed between the thighs. All the urine was collected for a few hours, but the syphon slipped out of position when the patient moved in bed, and the instrument ceased working.

It was difficult to secure the tubes in place without constricting their calibre. For this reason, I made them of hard rubber instead of soft, expecting to obtain the same results. I was disappointed. The water was raised over the bend of the tubes by the force of capillarity, but no partial vacuum being produced by its descent, the syphon did not start. The details of the experiment were varied in many ways without changing the result. Finally, I made the tubes of glass, so that the changes which took place within were exposed to view. In this way, I found that in hard tubes the water rose only along a narrow line at the lower aspect of the approximated surfaces. When the point *a*, (Fig. 1), the end af the small tube, was reached, instead of forming a drop, in descending the water spread out over the surface of the large tube in a thin layer which of course did not displace the air. The cause of failure was thus at once made apparent. In the system of soft tubes, on the other hand, the surfaces were equally distant at all points and the water was raised by capillary attraction in the form of a hollow cylinder, the sides of which coalesced at the end of the short tube in drops which in descending retained their form in virtue of the cohesion of their molecules.

The film of water on the surface of the hard tube must be converted into drops. In Fig. 1 is shown a section of the contrivance by which it was enabled to do this. The short, hollow cylinder closed at one end, (shown in the diagram,) was fitted to the extremity of the descending portion of the tubes. Perforating the bottom and extending upwards into the cavity of the cylinder, is a small tube which is continuous below with one of soft rubber. When the water is delivered by capillarity at *a*, (Fig. 1), if hard tubes are employed it descends, adhering to the sides of the cylinder as already described. The cylinder gradually fills until the water rises above the extremity of the upright tube. This tube, being small and the water entering from all sides, is entirely filled, the air is driven out and the syphon starts.

Having perfected the apparatus, I made no further use of it, because the study of the physical laws, which gave rise to these phenomena, had suggested to my mind a simpler method of reaching the same results. By these experiments, I had learned that, if the water were made to fill the tube, as small a quantity as four or five drops per minute, flowing continuously, were sufficient to start a syphon and keep it in constant operation. Since, as we have seen, a part of the urine is collected by any form of drain, it occurred to me, that this portion might be utilized to start the syphon and draw the remainder of the secretion after it. Fig. 2 shows a section of the instrument which I made in

FIG. 2.

FIG. 2. Double Chambered Drainage instrument tested experimentally.

the hope of accomplishing this result. I consists of one of Bozeman's drainage dilators divided into two cavities by a partition. The highest part of the upper chamber is connected by a tube with the lowest part of the inferior chamber. The walls of the instrument are perforated by openings leading from above into the upper and from all sides into the lower chamber. The same contrivance which I employed to cause the fluid to fill the tube in the self-starting syphon was fitted to the nozzle.

In the figure the instrument is represented as being tested experimentally. It is placed in a saucer of water, the air is excluded from the upper chamber by a soft rubber membrane, and water is being injected by means of a hypodermic syringe. By experimenting in this way I learned that the descent of about twenty drops of water through the soft rubber tube, was sufficient to partially exhaust the air in the instrument, and to cause the water in the saucer to rise and issue in a jet from the extremity of the tube connecting the two chambers. When in use the vesico-vaginal septum takes the place of the rubber membrane, the urine flowing directly from the bladder furnishes the power, and that which escapes into the vagina, and would otherwise be lost, is drawn from the lower into the upper chamber and flows out into the urinal.

Clinically, the instrument was only a partial success. At times all the urine was collected, at others none at all. The explanation of this, although it proved to be simple, puzzled me for a long time. In order to cause a rarification of the air in the upper chamber of the instrument, the urine must *descend* through the collecting tube. In constructing the drain I calculated upon a fall of at least four or five inches between the summit of the perinæum and the bed; but at times the urinal was nearly on a level with or even above the nozzle of the instrument. When this was the case, it is evident that the urine instead of flowing out would rise in the upper chamber, overflow the connecting tube, pass into the lower chamber and escape from the vagina. The fall from the bed to the floor is amply sufficient; but when this was tried it was found that the resistance afforded by the length and upward inclination of the tube between the patient and the edge of the bed, frequently caused the urine to take the easier avenue of escape between the surface of the instrument and the walls of the vagina.

THE PUMP.—Neither the urine collected by the instrument or raised by capillarity proved available to supply the small quantity of fluid necessary to start and keep the syphon in operation. There, however, remained another resource, water could be placed in a vessel on a table beside the bed, and allowed to flow drop by drop into the tube beyond the point where it bends over the edge of the bed. This brings us to the consideration of the pump. If the way has seemed long and this consummation delayed, be assured that you have reached it by a far shorter route than I. Only those experiments which serve to explain the final result have been described; a far greater number have been passed over in silence.

Fig. 3 represents the pump fitted up ready for use and Fig. 4, which is drawn on a larger scale, shows more minutely its more complicated parts. The glass cylinder (Figures 3 and 4) is closed at each extremity by a rubber stopper. A hard rubber stop-cock and a bent glass tube are fitted in perforations in the stopper at the upper end of the cylinder. The stop-cock is con-

FIG. 3.

FIG. 4.

FIG. 3. The pumb ready for use.

FIG. 4. The more complicated parts of the pump, shown more in detail, on a scale half the actual size. Length of cylinder 2⅛ inches: internal diameter of cylinder ¾ inch; internal diameter of glass syphon ⅛ inch.

nected by a soft rubber tube with one of metal, which is driven into an ordinary wooden bucket. The bucket, when filled with water, is intended to be placed upon a table of convenient height. The position of the cylinder is regulated by the length of the rubber tube, which serves to support it and at the same time to carry the water from the bucket. The bent glass tube (*a*, Fig. 4,) connects the cylinder with a soft rubber tube (*c*, Fig. 3,) which conveys the aspirating force to the place where the work is to be done. Perforating the lower stopper, is a glass tube (*b*, Fig. 4,) bent in the form of a syphon. The short arm and bend of the syphon lie altogether within the cavity of the cylinder, the long arm is continued downward by a soft rubber tube (*d*, Fig. 3,) which terminates in a vessel (preferably a foot-basin) placed upon the floor.

When the pump is working the water descends from the bucket into the cylinder. The quantity can be regulated with great accuracy by the stop-cock. The water rises above the bend of the syphon, then flows down the tube, and the cylinder is rapidly emptied. At the instant when the extremity of the short arm becomes uncovered, the bend and long arm of the syphon are full of water, which in descending draws a part of the air in the cylinder after it. The phenomena which follow, vary according as the soft tube *c*, Fig. 3, is open—allowing the air to enter the cylinder, or is immersed in a liquid, and are influenced by the rapidity with which the water flows from the bucket. When the tube is open and the supply of water is less than about two drachms per minute, the action of the pump is intermittent. All the water escapes from the long arm of the syphon while the short arm is still uncovered; the air rushes in and restores the equilibrium of the atmospheric pressure; the cylinder refills, and the series of events described is produced.* In order to lessen the length of the intermissions, I diminished the capacity of the cylinder by thickening the walls on two sides in such a manner as to convert the cylindrical into a quadrilateral space.†

If the supply of water from the bucket exceeds two drachms per minute, the action of the pump is continuous, and a constant current of air flows through the tube *c*, Fig. 3, when it remains open. Before all the water in the long arm of the syphon escapes, the rise of the fluid in the cylinder closes the extremity of the short arm, which is again uncovered by the removal of a

*The study of these phenomena is made more easy by using glass tubes instead of rubber.

†This change, although an improvement, is not essential. It necessitated the making of a mould at considerable expense. The mould is in possession of Messrs. John Reynders & Co., of New York, who also have exact models of all my instruments.

small quantity of water. In consequence of the alternate opening and closing of its upper extremity by the rise and fall of the fluid in the cylinder, the tube becomes filled by an endless series of minute cylinders of air and water.

When the tube *c* is closed by being immersed in a liquid the action of the pump is continuous, whether the supply of water from the bucket be great or small, the only difference being that the air in the cylinder is more rapidly exhausted in the former than in the latter case. As the rarification of the air proceeds, the water rises in the tube *c* until it flows over into the cylinder. After this occurs the pump acts like an ordinary syphon. The height to which the water can be raised, varies with, and is practically equal to, the distance of the cylinder from the floor.

From the above description of the pump, it will be seen that the principle involved in it is the same as that utilized in Sprengel's air-pump; but water is used to work the former, mercury the latter. There are pumps, as for example Bunsen's, in which the power is drived from the descent of water in tubes, but these require a large quantity to run them, and their use is for this reason confined to chemical laboratories, where the service pipe of water-works is at hand. A single bucket, holding three gallons of water, is sufficient to work the pump which I have described, rapidly enough to drain the vagina, or a suppurating cavity, continuously for twenty-four hours.

The disparity between the quantity of mercury used in Sprengel's pump and the water in Bunsen's, is due to a difference in the physical properties of the two fluids. In consequence of the great cohesive force of its molecules, and the fact that it does not moisten their surfaces, mercury in falling through tubes fills their calibre. Water, when in very small quantity, behaves differently. The tendency to remain in a mass or a thick layer due to cohesions of its molecules, is opposed by the attraction of he surfaces with which it comes in contact. This difficulty is overcome in Bunsen's pump by the excess of water used, which crowding through the tube fills its calibre. In my pump it is met in a way which has already been partly studied in connection with the self-starting syphon, and which we will now consider more carefully.

When a small quantity of water passes through the minute opening in the stop-cock, in consequence of the attraction of the glass, in descending it spreads out in a film upon the surface of the cylinder. If the end of the tube *b*, Fig. 4, were directly continuous with the bottom of the cylinder, the thin layer of water would extend downward along its innner surface. The thickness of this layer depends upon the rapidity with which the water flows from the stop-cock. If it reaches a certain thickness, varying with the diameter of the tube, the opposing surfaces of the water unite, the calibre of the tube is filled, and the exhaustion of the air in the cylinder begins. But when the tube is sufficiently large not to be obstructed by fragments of mucus, pus, and other solid particles, which must pass through it, the quantity of water required is too great to be conveniently supplied. By causing the water to traverse the bent tubes Fig. *b*, 4, this difficulty is overcome. Whatever the size of the tube and however small the quantity of water, the fluid will fill the short arm of the syphon, because it rises from below upward; it, therefore, enters the bend and descending portions of the tube, not as a thin layer but as a solid cylinder. The causes which tend to prevent the water, which has been moulded into a cylinder, from again flattening out into a thin layer in passing through the transverse and descending portions of the tube are the following:

1. The imperfect fluidity of water. Force is required to effect the re-arrangement of the molecules of a liquid necessary to transform a mass into a thin layer, and *vice versa*. For this reason, water tends to retain the form that has been imparted to it.

2. The attraction of the glass tending to cause the fluid to flatten out in a thin layer on its surface, is now in great part neutralized, because it is exerted in all directions.

3. The cylinder of water is in motion.

There is, however, a limit to the size of the tube that can be used with a very small supply of water. In tubes which exceed this limit, its weight causes the water, as it traverses the transverse portion, to sink down upon the lower surface of the tube.

I have determined experimentally the largest tube that can be used with the smallest quantity of water that can be made to flow through the stop-cock. It is one-eighth of an inch in diameter. Fortunately, this is large enough for all practical purposes, and is the size which I use. A somewhat larger tube may, however, be employed if the supply of water is moderately increased.*

In using the pump, there are several practical points worthy of attention. When the stop-cock is so regulated as to allow only a few drops a minute to pass through it, the aperture is very small and will become obstructed if the water holds any solid particles in suspension. When this is the case, the water must be filtered. The best way to do this is to fasten a piece of absorbent cotton or sponge over the end of the faucet (see Fig. 3,) where it projects into the cavity of the bucket. The pump works more evenly when the lower extremity of the tube *d*, Fig. 3, is not immersed in water. It, therefore, should not reach to the bottom of the vessel placed beneath it on the floor. The rubber bag of a fountain syringe, or, in fact, any vessel which will hold the necessary quantity of water may be used instead of the bucket, but the latter is cheaper and generally more convenient, because one can usually be found in every house.

APPLICATION.—The cheapness, simplicity and convenience of the pump adapt it for a variety of medical and surgical uses. The most important of these depend upon its power of slow and continuous action, which noiselessly goes on while the patient sleeps. The pump may also be used in cases where a more rapid exhaustion of the air is required. We have therefore in this simple instrument, not only a means of draining the vagina, but the bladder, the pleural cavity when it contains pus, Douglass' *cul de sac* after laparotomy, abscesses, suppurating wounds, and, in fact, a means of removing pus, decomposing secretions

---

* By reference to Fig. 2, it will be seen that the cylinder of the pump with its accessories is almost identical with the contrivance which I used in the self-starting syphon in order to make the water fill the tube. There is this important difference, however, the upright tube extending up into the cylinder is bent over in the form of a syphon. I was led to make this improvement by observing in experiments with instruments like that shown in Fig. 2, that when water first ascended and then descended in a tube, no special contrivance was necessary to make the fluid fill its calibre.

and deleterious discharges from dependent localities, and natural or pathological sacs. At times it is also desirable to combine with this perfect drainage continuous or periodical irrigation, and this is also made easy because the pump removes the antiseptic solution so that it does not wet the bed and the patients' clothing. Besides these, there are other applications not susceptible of classification.

DRAINAGE.—Certain alterations must be made in ordinary drainage tubes when they are used in conjunction with the pump. These become necessary in order to prevent the suction from being exerted upon the surrounding tissues, which, besides being itself objectionable, may cause them to block up the openings in the tube and defeat the end in view. In order to obviate this difficulty, the tube must be so arranged that its distal extremity will communicate with the atmosphere. This may be done in two ways, either of which may be adopted according as circumstances render the one or the other convenient. The tube may be bent upon itself, the two ends occupying the mouth of the cavity and the loop the lowest part; or the tube may traverse the cavity and reach the surface of the body through openings more or less distant from each other. The discharges are admitted by lateral apertures in the loop and distal portion of the tube. No perforations should be made in that part of the tube connecting the loop with the pump.

When the pump is working, a constant current of air is drawn through the drainage tube and there is no suction upon the tissues. As soon as an appreciable quantity of fluid collects at the lowest part of the cavity, it passes through the openings in the loop and interrupts the current of air by filling the interior of the tube. The full force of the pump is now acting upon the pus, which is at once borne up with the current of air and removed from the body.

At first sight it would seem that this current of air passing through the tube might carry into the cavity germs or other deleterious matters, but this danger is only apparent. The end of the tube is covered and the air is filtered by the dressings. Even if this were not the case, there would be little danger, because the air does not pass through the cavity, but through the tube and, instead of coming in contact with the tissues, the tendency is in the opposite direction—from the walls of the cavity into the tube.

This duplex arrangement of the drainage tube makes it convenient for irrigation; the nozzle of a fountain syringe may be inserted into its distal extremity, and the tube going to the pump compressed with the fingers. The cavity is in this way filled with fluid, which will be carried away by the pump when the fingers are removed. Instead of this periodical irrigation, a continuous current may be made to pass through the tube; but the solution does not come in contact with the tissues, it merely traverses the tube.

Besides these soft rubber drainage tubes, which may be improvised, Messrs. John Reynders & Co., of New York, have

FIG. 5.

FIG. 5. Vaginal drainage tube, ⅓ actual size. Dimensions: Length 4 inches; transvers diameter of Loop 1⅜ inches; internal diameter of tube ⅛ inch.

made for me several of hard rubber to be used in cases where rigid tubes can be employed with advantage. The tube shown in Fig. 5 is intended to drain the vagina when a vesico-vaginal fistula exists. The loop is made large in order to render the instrument self-retaining, and the limbs of the tube, which are se-

FIG. 6.

FIG. 6. Vesical drainage tube. Dimensions the same as the vaginal instrument, except the transverse diameter of the loop which is ¾ inch.

curely fastened together, are bent downwards near their anterior extremities in order to conform to the axis of this part of the vagina. The distal part of the tube should be lengthened so as to extend outside the vulva by a soft rubber tube fitted on at *a*. Connection with the pump is made at the other extremity of the tube. The tube shown in Fig. 6, is intended to drain the male bladder after perinæal section. It differs from the one just described only in the size of the loop, which is made small so as to be readily introduced through the opening into the bladder. A drainage tube for Douglass' *cul de sac* after laporotomy is shown in Fig. 7.

FIG. 7.

FIG. 7. Peritoneal Drainage Tube. Dimensions: Length 5 to 7 inches; transverse diameter of loop ¾ inch; internal diameter of tube ⅛ inch.

The likeness between this latter and those which precede it is so close that a special description is unnecessary. In fact, all three are identical in principle with the soft rubber tubes already described, and differ from each other only in so far as is necessary to conform to the anatomical peculiarities of the various localities which they are intended to drain.*

CONTINUOUS IRRIGATION.--In some other cases, drainage is not needed, but advantage is derived from the application of warm water to the affected parts, and here again the pump is useful because the fluid must be conducted away so as not to wet the bed and the patient's clothing. Figure 8 shows a support for holding in position tubes to be used for continuous irri-

*The contact of the urine causes the salts of lime to be deposited in the interior and on the surface of the tubes; but the deposit is readily removed by the action of hydro-chloric acid, which does not injure the hard rubber.

gation of the external ear. A rubber stopper, in which two perforations have been made, is fitted in a broad ring supported by a bent rod of copper attached to a thin metal plate. The plate is fastened to the head above the ear by a bandage or a ribbon. The tubes are passed through the perforations in the stopper, one of them conveys warm water to the ear, the other, which is attached to the pump, carries it away. The quantity of

FIG. 8.

FIG. 8. Support for holding tubes to be used for continuous irrigation of the external ear, (half actual size).

water going to the ear may be regulated by a clamp, but a stop-cock is more convenient. The water sinks into the external auditory canal and rises in the concha, (which forms a convenient basin into which both tubes dip,) to a height regulated by the position of the efferent tube.

REMOVAL OF FLUIDS FROM AND in all of its applications, which have been thus far considered, a small quantity of water is used to work the pump continuously for a number of hours. In those which follow, a more rapid exhaustion of the air, continued for a shorter time, is required and the water must be allowed to enter the cylinder more rapidly.

IRRIGATION OF SURFACE AND CAVITIES.—When the pump is working in this way, it may be employed to empty abcesses which have been opened, and to remove fluid which has been injected into cavities in order to wash them out. A glass tube, inserted into the rubber tube going to the pump, is a convenient instrument for this purpose.

In Fig. 9, is shown a more important instrument which combines in one the offices of an irrigator and a sponge for the removal of blood during an operation, or of pus in the treatment of wounds. It is a glass tube bent upon itself forming a loop below.

FIG. 9.

FIG. 9. Tube for irrigation and drainage of bleeding or suppurating surfaces, ⅓ actual size. Dimensions: Length 3 to 9 inches; transverse diameter of loop ¾ inch; internal diameter ⅛ inch.

Its two extremities are slightly separated so as to allow a rubber tube to be fitted upon each. One of these tubes is connected with the vessel holding the antiseptic solution, the other goes to the pump. At the central part of the loop is a small glass tube or nozzle, half an inch long. The solution passes constantly down one limb of the tube, across the loop up the other limb, and out through the pump; the quantity which enters the system of tubes is regulated by a hard rubber stop-cock. In traversing the loop, the solution becomes mixed with the blood or pus, and together with a considerable quantity of air is drawn through the nozzle. The solution does not pass through the nozzle, because it is acted upon by the full force of the pump, which is greater than the pressure from behind of the fluid descending in the tube.

When, however, the soft rubber tube going to the pump is compressed with the fingers, the fluid flows out as from the nozzle of an ordinary irrigator. As soon as the surface is inundated, the solution may be taken up by simply removing the pressure and allowing the aspirating action of the pump to go on.

Besides the advantages derived from irrigation, this duplex arrangement renders the instrument preferable to a single tube, because the solution, by diluting the blood or pus, increases its bulk. This is important, for, when a small quantity of liquid is borne upwards by a current of air, it is quickly blown into bubbles; these collapse, and the resulting layer of fluid adhering to the glass sinks to the bottom; a succession of bubbles rise and disappear, but the drop constantly returns to the lower extremity of the tube.

Combining as it does the direct and recurrent stream, the instrument is also very convenient for washing out cavities and may be used as a vaginal *douche*. When disconnected from the tube carrying the antiseptic solution, the double tube has an advantage over the single one, when used to remove fluid which has been injected into a cavity. The open extremity of the straight tube is liable to be closed by the tissues when they are soft and yielding, as for example in the peritoneal cavity. In the double tube this difficulty is overcome in the same way as in the drainage tube; free communication with the atmosphere being provided, the force of the pump is exerted only upon the liquid in the interior of the tube. When the instrument is used in this way, it is better to make it without the nozzle and allow the fluid to enter through a large perforation at the middle of the loop.

For washing out the uterus and applying iodine, carbolic acid, and astringent or antiseptic solutions to the mucous membrane lining the interior of the womb, the pump will prove useful. Hitherto, there has been an element of danger in procedures of this sort which has deterred some from resorting to them. Fluid when injected into the uterine cavity is liable to, and sometimes does, pass out through the Fallopian tubes into the peritoneal cavity. Considerable ingenuity has been exercised by the profession in endeavoring to overcome this difficulty and a number of double current tubes have been devised in order to afford to the fluid a free escape from the uterus.

In these instruments, the fluid is injected into the uterus and the uterine cavity is more or less forcibly distended. Fig. 10 illustrates a new method of introducing and applying the solution to the mucous membrane. A nozzle *a*, Fig. 10, connected with the pump, is fixed to the efferent tube of Bozeman's double current catheter, a soft rubber tube dipping into the antiseptic solution is fitted to the efferent tube and, in order to exclude the air, the fenestrated part of the instrument is enclosed in a narrow bottle. Rarification of the air in the bottle being produced by the pump, the solution rises in the efferent tube, issues in a jet from its

FIG. 10.

FIG. 10. Diagram illustrating the principle involved in a new method of making applications to the interior of the uterus.

extremity, and the external tube becomes filled by the escaping fluid which flows out through the pump. When in use, the uterus takes the place of the bottle and excludes the air and the solution comes in contact with the mucous membrane opposite the fenestra on each side.

The most obvious advantage derived from this method of irrigation is, that the returning fluid is carried off by the pump and does not soil the bed or the patient's clothing. But more important than this, a minus in place of a positive pressure is produced in the uterus and, instead of the solution being forced out through the Fallopian tubes, there is acting in the opposite direction a strong suction force tending to empty their contents. When we consider the relations of the Fallopian tubes to the uterine cavity, we find that their orifices lie opposite to the lateral openings in the instrument. They are also stretched open, and the folds of the mucous membrane effaced by the pressure of the instrument, which, to a greater or less degree, distends the uterine cavity.

The tendency to collapse of the part of the tubes which traverses the walls of the uterus is moreover opposed by the unyielding character of the surrounding tissue.

I am now making a uterine applicator which will work upon this principle, and hope, at least in favorable cases, to remove pus from the Fallopian tubes when sal-pingitis complicates endometritis, and at the same time apply appropriate medication to the lining membrane of the uterus.

## Figures and Tables

**Fig. 1. f1:**
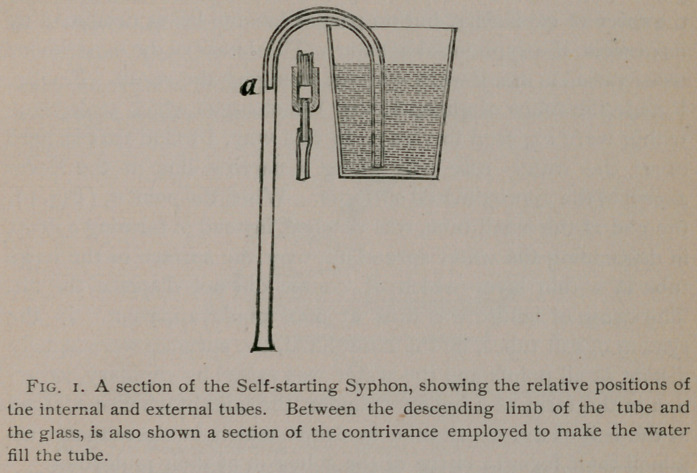


**Fig. 2. f2:**
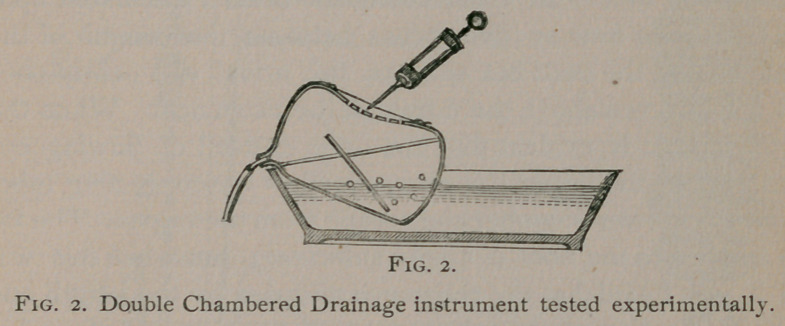


**Fig. 3. f3:**
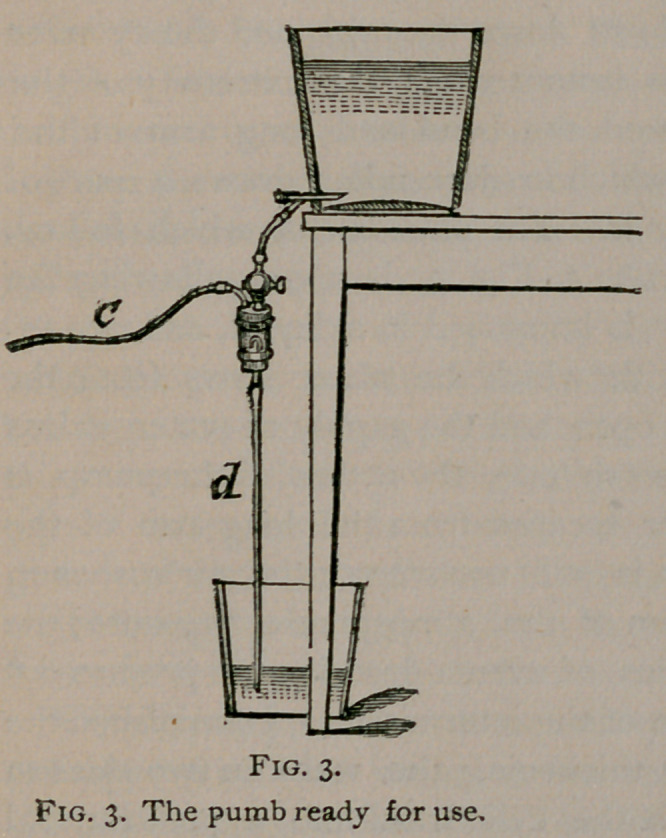


**Fig. 4. f4:**
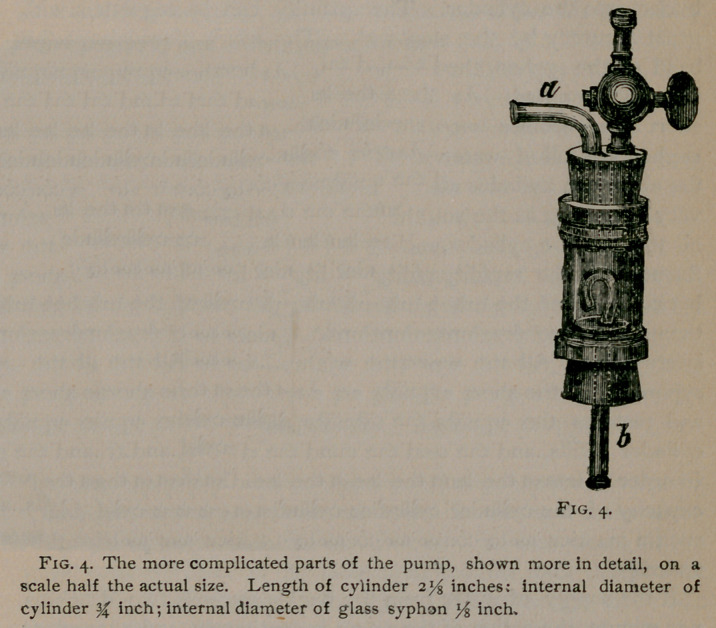


**Fig. 5. f5:**
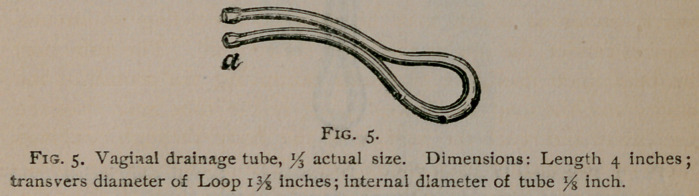


**Fig. 6. f6:**
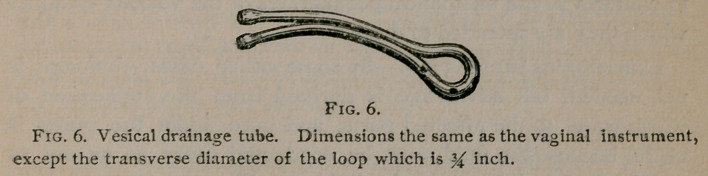


**Fig. 7. f7:**
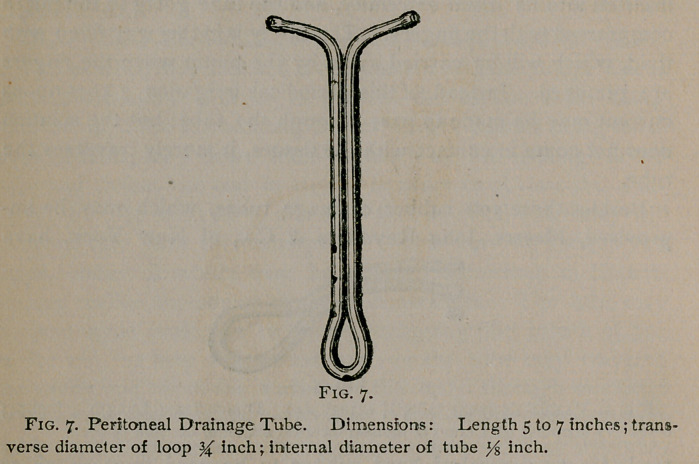


**Fig. 8. f8:**
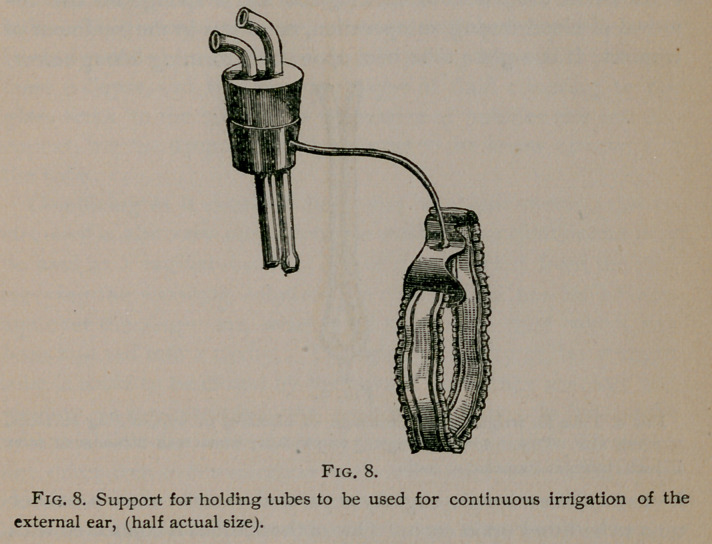


**Fig. 9. f9:**
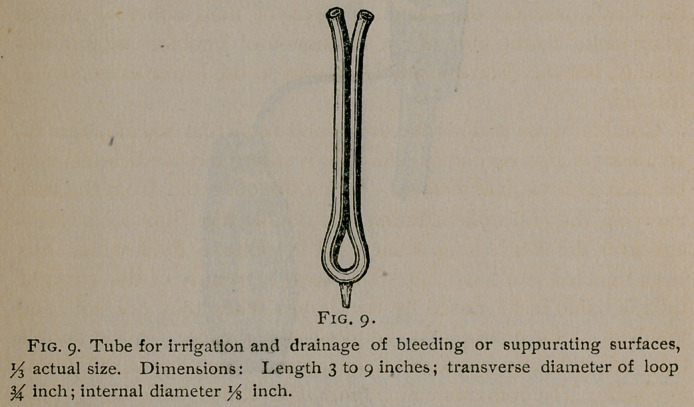


**Fig. 10. f10:**